# Targeted Protein Degradation: Advances, Challenges, and Prospects for Computational Methods

**DOI:** 10.1021/acs.jcim.3c00603

**Published:** 2023-08-21

**Authors:** Barmak Mostofian, Holli-Joi Martin, Asghar Razavi, Shivam Patel, Bryce Allen, Woody Sherman, Jesus A Izaguirre

**Affiliations:** † OpenEye, Cadence Molecular Sciences, Boston, Massachusetts 02114 United States; ‡ Laboratory for Molecular Modeling, Division of Chemical Biology and Medicinal Chemistry, Eshelman School of Pharmacy, University of North Carolina, Chapel Hill, North Carolina 27599 United States; ¶ ENKO Chem, Inc, Mystic, Connecticut 06355 United States; § Psivant Therapeutics, Boston, Massachusetts 02210 United States; ∥ Differentiated Therapeutics, San Diego, California 92056 United States; ⊥ Atommap Corporation, New York, New York 10013 United States

## Abstract

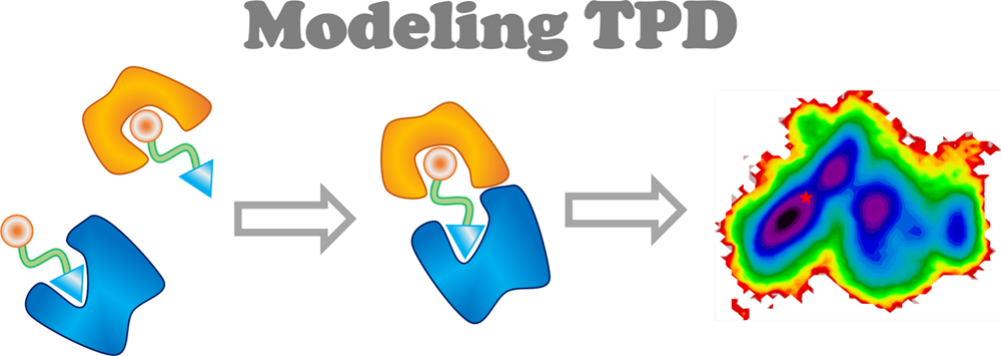

The therapeutic approach of targeted protein degradation (TPD) is gaining momentum due to its potentially superior effects compared with protein inhibition. Recent advancements in the biotech and pharmaceutical sectors have led to the development of compounds that are currently in human trials, with some showing promising clinical results. However, the use of computational tools in TPD is still limited, as it has distinct characteristics compared with traditional computational drug design methods. TPD involves creating a ternary structure (protein–degrader–ligase) responsible for the biological function, such as ubiquitination and subsequent proteasomal degradation, which depends on the spatial orientation of the protein of interest (POI) relative to E2-loaded ubiquitin. Modeling this structure necessitates a unique blend of tools initially developed for small molecules (e.g., docking) and biologics (e.g., protein–protein interaction modeling). Additionally, degrader molecules, particularly heterobifunctional degraders, are generally larger than conventional small molecule drugs, leading to challenges in determining drug-like properties like solubility and permeability. Furthermore, the catalytic nature of TPD makes occupancy-based modeling insufficient. TPD consists of multiple interconnected yet distinct steps, such as POI binding, E3 ligase binding, ternary structure interactions, ubiquitination, and degradation, along with traditional small molecule properties. A comprehensive set of tools is needed to address the dynamic nature of the induced proximity ternary complex and its implications for ubiquitination. In this Perspective, we discuss the current state of computational tools for TPD. We start by describing the series of steps involved in the degradation process and the experimental methods used to characterize them. Then, we delve into a detailed analysis of the computational tools employed in TPD. We also present an integrative approach that has proven successful for degrader design and its impact on project decisions. Finally, we examine the future prospects of computational methods in TPD and the areas with the greatest potential for impact.

## 1 Introduction

### 1.1 Historical Overview of Degradation

Protein degradation plays a crucial role in cellular regulation.^1^ The 26S proteasome is the primary enzyme in the ubiquitin-dependent protein degradation pathway, which is the main focus of this Perspective. The proteasome, a nanomachine that relies on energy, breaks down proteins that are covalently “tagged” with ubiquitin by an E2 ligase. This ligase usually participates in a larger macromolecular assembly responsible for identifying proteins to be targeted for degradation through a degron recognition motif. The ubiquitin–proteasome pathway is vital for controlling protein levels in cells and, therefore, essential for life.^2^ Since the early 2000s, several researchers have endeavored to exploit this pathway using molecules that induce proximity and can be leveraged for therapeutic purposes to degrade a protein of interest (POI).^3−6^ A degradation strategy offers multiple advantages over small molecule inhibitors, including targeting nonfunctional sites on the POI and the catalytic mechanism by which it operates. While traditional small molecule drug discovery has primarily focused on directly controlling protein activity, harnessing the endogenous protein destruction machinery within cells to selectively degrade key drivers of human diseases holds great potential for drug discovery.

The discovery of cells’ ability to degrade proteins through a nonlysosomal pathway dates back to the early 1980s.^7^ Although the mechanism was initially unclear, researchers soon identified a small regulatory protein called ubiquitin, which was covalently attached to the protein before degradation. Subsequently, it was found that three enzymes (E1, E2, and E3) played a role in the ubiquitination process.^8^ The large protein assembly (20S proteasome, part of the 26S proteasome) responsible for protein degradation was characterized^9^ and crystallized^10^ in the 1990s. These groundbreaking discoveries inspired drug researchers to investigate the possibility of inducing the proximity between a protein of interest and an E3 ligase, leading to growing efforts in academia and industry to develop molecules capable of harnessing degradation biology for therapeutic purposes. The first heterobifunctional degrader molecule was reported in 2001^11^ and in 2019, the first designed degrader molecule entered human clinical trials.^12^ This progress has sparked a surge of interest and investment in the field of targeted protein degradation (TPD). Initial research efforts in TPD focused on well-validated cancer targets such as estrogen receptor (ER)^13^ and androgen receptor (AR).^14^ However, the TPD strategy extends beyond specific indications or therapeutic areas.^15^ As a result, TPD research now covers a wide range of targets and therapeutic areas, including notoriously challenging targets like KRAS^16^ and MYC,^17^ as well as lesser-known targets such as IRAK4,^18^ STAT3,^19^ and Tau.^20^

The process of targeted protein degradation by a degrader molecule consists of several steps that can impact the degradation efficiency. Some of these steps resemble those of traditional small molecule therapeutics, while others are unique. For instance, like traditional small molecules, a degrader must possess an adequate solubility and permeability to reach the protein of interest (POI). However, the mechanistic steps diverge once the degrader reaches the POI. First, TPD is a catalytic process in which a single molecule can cause the degradation of numerous POI copies.^21,22^ The process commences when the degrader induces proximity between a POI and an E3 ligase by forming ternary complexes.^23^ These complexes can involve non-native interactions between the two proteins as the therapeutic strategy often involves repurposing the E3 ligase to act on non-native substrates. The strength and nature of these protein–protein interactions play a crucial role in degradation efficiency.^24^ Moreover, the ternary complex is typically flexible, meaning that static representations might be inadequate for correlating structure to function.^25^ The formation of the ternary complex enables the ubiquitination of the POI, which generally involves ubiquitin-conjugating enzymes (E2s).^26^ However, not all ternary complexes lead to the same level of POI ubiquitination due to the spatial and kinetic aspects of the ubiquitination process. Lastly, once ubiquitinated, the POI is prepared for degradation by the proteasome.^27^

Solving crystal structures of ternary complexes has been achieved,^23^ but converting these structures into actionable designs is not a simple task. Empirical rules created for traditional small molecules may not be readily applicable as most heterobifunctional degraders have a molecular weight significantly above 500 Da. Computational chemistry tools, such as small molecule docking, are not designed to simultaneously bind a degrader molecule and two proteins. Additionally, the relationship between the ternary structure and degradation efficiency remains unclear. Some researchers argue that more rigid linkers are better,^28^ while others suggest that flexibility is superior.^29^ The answer is likely dependent on the specific system’s nuances, with numerous factors contributing to the design of an effective degrader molecule.

### 1.2 Heterobifunctional Molecules as Therapeutics

**Figure 1:**
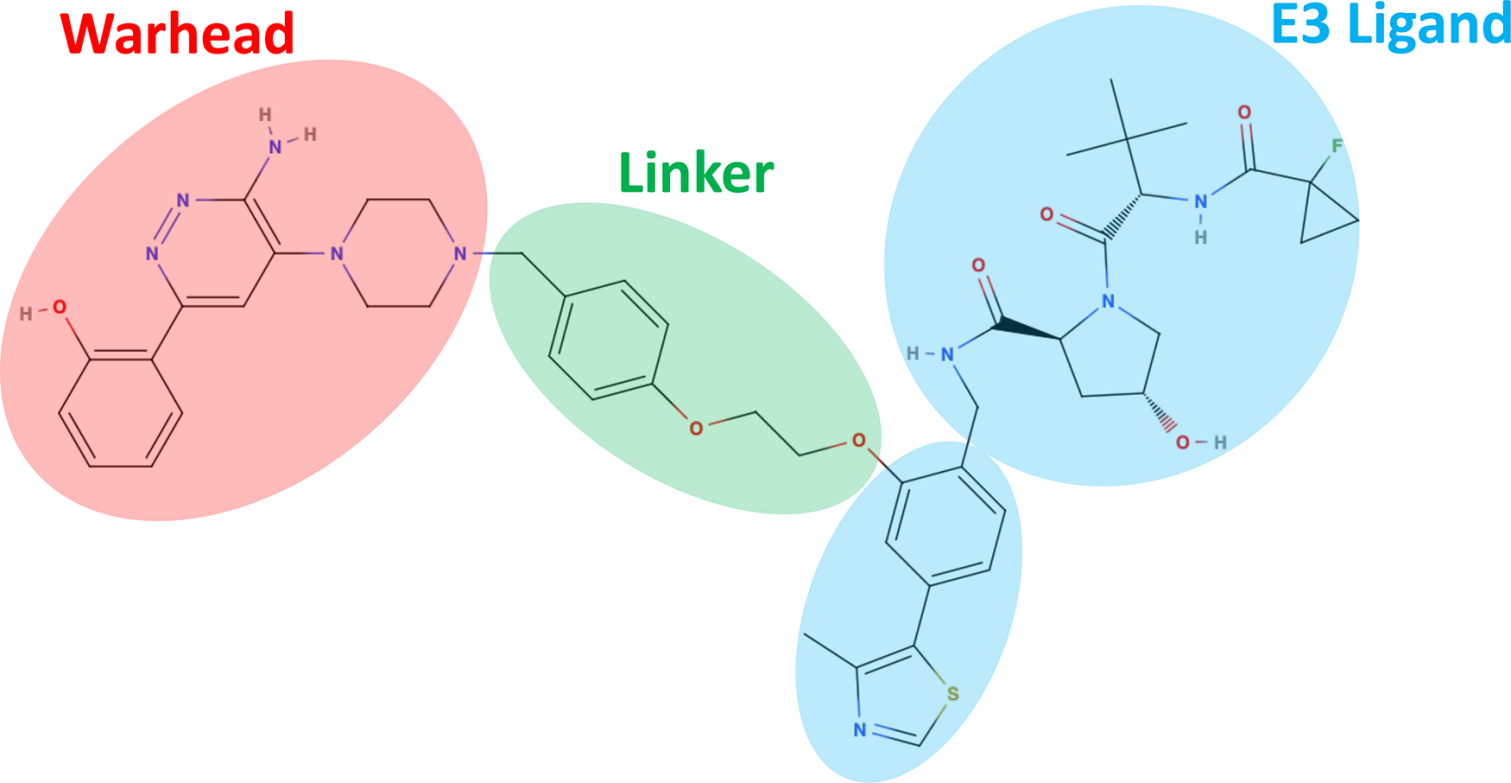
Example of a heterobifunctional degrader molecule. The structure of ACBI1 is depicted, a prominent degrader of SMARCA2 that recruits von Hippel–Lindau (VHL) E3 ligase. The warhead (red), linker (green), and E3 ligand motifs (blue) are highlighted, illustrating the typical composition of a degrader molecule.

Therapeutic molecules for targeted protein degradation can generally be classified into “glues” and “heterobifunctionals”, although in reality there is a spectrum between these two extremes. Glues resemble traditional small molecules in shape and size that, upon binding, function as “adhesives” and convert the target protein into a so-called “neo-substrate” for the E3 ligase. Conversely, heterobifunctional degraders, as visualized in Figure 1, are larger and consist of two protein-binding components connected by a linker motif, usually inducing a more flexible ternary complex. In this Perspective, we focus primarily on heterobifunctional degrader molecules.

In terms of nomenclature, we will refer to the molecular motif binding the protein of interest as the “warhead”, the motif binding the E3 ligase as the “E3 ligand”, and the “linker” connecting the two motifs. This topology presents both opportunities and challenges in the therapeutic design. On one hand, it is possible to rapidly design degrader molecules by connecting a known warhead to an E3 ligand using a flexible linker, as demonstrated in numerous examples.^30−33^ However, the linker’s nature can significantly affect degradation, likely due to a combination of factors that will be discussed in greater detail below. As a result, while hit finding can be relatively straightforward in TPD programs when there are known POI warheads, designing a degrader remains a challenging task.

First, enhancing properties such as solubility and permeability is often more challenging for heterobifunctional degraders than for traditional small molecules. This is due to the lack of substantial data and the innate complexity of these larger, more flexible molecules. Moreover, connecting chemical structures to degradation is difficult, as binding is necessary but not sufficient to induce ubiquitination, and enzymatic deubiquitination may interfere. Additionally, degraders could bind to each protein-binding partner individually leading to the concentration-dependent hook effect, i.e., the formation of binary complexes that compete with the productive ternary complexes and thus negatively impact the degrader potency and also complicate toxicological assessments.^34−36^ Furthermore, there are only a few computational tools specifically designed for predicting and designing the properties of heterobifunctional molecules. To predict the activity of these molecules, it is beneficial, and sometimes essential, to consider factors beyond binding, such as the conformational landscape of the ternary complex ensemble, which is crucial for understanding functionality. Lastly, while several molecules based on heterobifunctional design are currently in clinical trials, there are no approved heterobifunctional degraders. As a result, there is limited information about potential toxicities and other liabilities associated with this emerging class of molecules.

### 1.3 Traditional Computational Approaches are not Suited to Heterobifunctional Molecules

Targeted protein degradation necessitates a novel approach to small molecule drug discovery, shifting from static structures that function primarily via occupancy-driven pharmacology to conformational stabilization within a complex free energy landscape to achieve catalytic turnover. X-ray structures are often inadequate for understanding structure–activity relationships (SAR), given the dynamic nature of the ternary complex and its implications for downstream ubiquitination. As previously mentioned, the warhead–linker–ligand configuration of heterobifunctional molecules, along with the importance of the ternary structure, poses challenges for traditional computational methods.

For instance, conventional small molecule docking tools can be used to position the warhead and E3 ligand to the POI and E3 ligases, respectively, but they are insufficient for predicting ternary structures. Likewise, traditional protein–protein docking tools do not consider small molecule sampling in conjunction with global (translational and orientational) protein sampling. In essence, predicting the ternary structure combines some of the most challenging aspects of both small molecule docking and protein–protein docking into one problem, although it is possible to simplify the search problem in certain cases by applying constraints.

Predicting other drug-like properties, such as solubility, permeability, bioavailability, and clearance, also presents challenges. Currently, there are minimal data available to create machine learning (ML) or quantitative structure–activity relationship (QSAR) models, and even if such data existed, the model-building methods might need to change. For example, fingerprint representations may be insufficient for constructing meaningful predictive models, and the quantity of data required to build high-quality models might be significantly larger for heterobifunctionals due to their size and conformational flexibility. Furthermore, traditional physiochemical properties might not directly inform project decisions, given the complex, multistep catalytic process. While the scarcity of data will improve over time, it is difficult to predict when enough information will be available to make reliable predictions on various properties for heterobifunctionals.

### 1.4 Assays for TPD

TPD shares some similarities with traditional drug discovery such as the necessity of binding, but it also exhibits numerous differences. TPD calls for a more diverse approach (both computationally and in the wet lab) utilizing various tools and unconventional ways of conceptualizing the problem. The lack of a direct correlation between binding and degradation necessitates that cellular assays be conducted earlier in the process and serve as a primary assay. Additionally, permeability can sometimes fall below the detectable limit by using traditional small molecule methods, further emphasizing the importance of early cellular assays.

Since the primary objective of TPD is to reduce protein levels, it is crucial to monitor these levels within the cell, typically through proteomic techniques. This monitoring becomes central to the project rather than just being part of exploratory biology. In fact, due to its catalytic nature, TPD demands a new way of thinking about target product profiles (TPPs), as the event-driven degradation mechanism can still exhibit high efficacy at lower doses compared to the occupancy-driven pharmacology of traditional inhibitors.

The intricacies of TPD suggest that successful research groups in this area will benefit from a more diverse workforce and the proper integration of various skill sets. Further details on TPD assays can be found in Section 2.

### 1.5 Overview of This Perspective

Although TPD research has thus far been successful without substantial computational investments, the development and deployment of appropriate tools can have a significant impact. Emerging examples show that traditional computational tools are being adapted for TPD applications, such as ternary complex docking, which will be covered in this Perspective. However, in our opinion, the most significant impact will stem from new techniques specifically designed for TPD applications, such as molecular simulations of various TPD steps, which we will discuss in detail.

To develop new tools, it is crucial to understand the physical steps associated with TPD. We will start by examining the targeted protein degradation process and the range of experimental assays used to explore it. Subsequently, we will discuss how each physical step can be modeled using different computational approaches, with a focus on predicting properties like solubility and permeability, multiscale simulations of various steps, and mathematical modeling of degradation events. As the ultimate goal of TPD research is to design innovative therapeutics, we will explore what an integrative design process for heterobifunctional degraders might entail, showcasing some recent results that demonstrate the impact of computational methods in degrader design and the value of integration with experiments. Lastly, we discuss potential areas for future investment and impact.

## 2 Overview of the TPD Process—From Administration to Protein Degradation

The TPD process involves a series of steps, all of which must occur for successful degradation. We describe the process from when a drug enters the body to when the targeted protein is degraded, with relevant assays interspersed.

**Figure 2:**
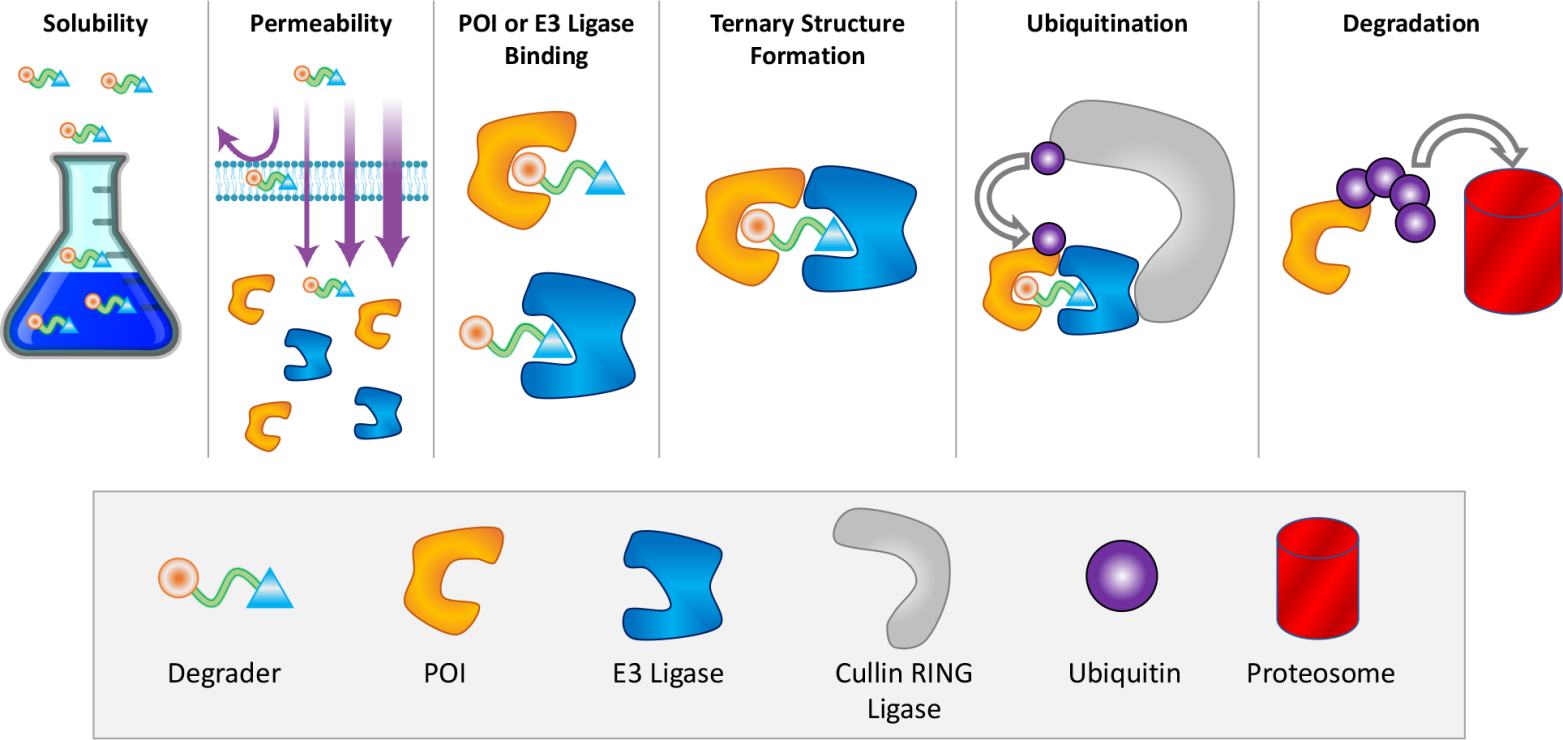
Physical steps of the TPD process. A degrader must be soluble in aqueous environments for its systemic circulation and distribution (“Solubility”), but it should also be lipophilic enough for passive transport into the cell (“Permeability”). Inside the cell, the degrader binds through its warhead to the target protein of interest and through its E3 ligand motif to an E3 ligase (“POI or E3 Ligase Binding”), yielding a so-called ternary complex (“Ternary Structure Formation”) that induces the “Ubiquitination” of the POI in a supramolecular assembly (e.g., the Cullin–RING ligase). “Degradation” is then achieved by the cell-innate ubiquitin–proteasome pathway.

Figure 2 shows a graphical depiction of the primary steps involved in the TPD process, from administration of a heterobifunctional degrader to theproteasomal degradation event. For a molecule to be efficacious as a drug it must reach the target of interest. For a degrader molecule this still holds true, although the amount required is likely to be significantly lower. The molecule must be soluble to get distributed in the blood and permeable enough for at least one copy of the degrader to get into the cell. Once in the cell, the molecule must reach the protein of interest, bind to the two partners (POI and E3), and induce ubiquitination. Compared with traditional small molecule drugs, degraders need a lower concentration in the cell because they operate by an event-driven mechanism.^21,22^ Thermodynamically, the order of operations does not matter in terms of binding to the POI or the E3 ligase first, although kinetically, there may be differences.

A variety of different experimental techniques and assays have been developed to characterize each of the TPD steps. As summarized in Table 1, some of the most commonly used assays can be broadly categorized into (1) target engagement assays, which are designed to measure the ability of a degrader to bind to the POI or E3 ligase, (2) ternary complex formation assays, which focus on evaluating the formation of the ternary complex, and (3) functional cellular assays, which evaluate the ability of a degrader to induce degradation of the target protein and its downstream effects on cellular function. A combination of these assays is often employed to characterize degraders, assess their specificity, and determine their potential therapeutic utility in discovery projects. In the following paragraphs, we discuss these and several other promising techniques and the caveats associated with TPD projects.

As with the development of any small-molecule drug, monitoring biopharmaceutical properties, e.g., bioavailability and pharmacokinetics, during the discovery of degraders is crucial to guide the decision-making process and to ensure the development of active and potent degraders. Several studies have offered insight into degrader metabolism and pharmacokinetics,^53−55^ concluding, based on solubility, biotransformation, and elimination data as well as on nonenzymatic stability analysis, that heterobifunctional degraders may well achieve acceptable drug metabolism and pharmacokinetics (DMPK) properties, although optimization strategies are required.

**Table 1:** Summary of Assays and Techniques That Have Been Employed for Targeted Protein Degradation

Category	Assay^a^	Readout	References
Target engagement assays	Surface plasmon resonance (SPR)^v^	Binding affinity and kinetics through changes in light reflection	(28,37−39)
	Isothermal titration calorimetry (ITC)^v^	Binding affinity, stoichiometry, thermodynamic quantities from heat generation/consumption	(28,40−43)
	Fluorescence polarization (FP)^v,c^	Binding affinity and selectivity by changing fluorescence signal	(28,37,41,43−45)
Ternary complex formation assays	Time-resolved Förster resonance energy transfer (TR-FRET)^v,c^	Formation of ternary complex by changes in fluorescence intensity	(28,39,45)
	Amplified luminescent proximity homogeneous assay (ALPHA)^v,c^	Ternary complex detection through luminescence signal	(28,38,40,41)
	Proximity ligation assay (PLA)^c^	Detection of ternary complexes by fluorescence or luminescence	—
Functional cellular assays	Western blot^v^	Quantification of target protein levels by specific antibodies	(28,37−47)
	CRISPR/Cas9-mediated gene editing^c^	TPD effects compared to genetic loss of the target protein	(28,41,48,49)
	Cell-based phenotypic assays^c^	Biological consequences of degrading the target protein	(50−52)

a The superscripts indicate if the corresponding experiment is usually performed *in vitro* (^v^) or as a cell-based assay (^c^).

Favorable aqueous solubility and membrane permeability are key attributes in the development of therapeutics^56^ and their mutual interplay, with reference to drug delivery and distribution, is well known.^57,58^ The correct estimation of these properties is critically important for ADMET (Absorption, Distribution, Metabolism, Elimination, Toxicity) screening and lead optimization, which is discussed in greater detail in Section 3.1 in the context of predicting and modeling different aspects of degraders. Importantly, the assessment of oral degrader absorption is more complicated than that of small-molecule drugs because, similar to macrocyclic or peptidomimetic compounds, degraders exhibit a certain “chameleonic behavior” in response to different environments, enabling them to passively permeate cell membranes and, yet, be sufficiently dissolved under aqueous conditions.^59,60^ Due to their relatively large structure, heterobifunctional degraders are assigned to the so-called “beyond rule of five” (bRo5) chemical space,^61,62^ i.e., the accurate estimation of their properties is strongly hampered. Therefore, recent lessons learned on the molecular features of bRo5 compounds will prove highly valuable for degrader design.^63−66^

A variety of experimental physicochemical profiling methods^67,68^ are usually applied to characterize properties such as polarity, lipophilicity, ionization, or stability, which are major determinants of a compound’s bioavailability.^69^ For instance, partition assays or reverse-phase HPLC^70,71^ measure the lipophilicity, usually reported as the octanol–water partition coefficient, Log P, i.e., the equilibrium distribution of a compound between water and octanol, or, as the distribution coefficient, Log D, for ionizable species, respectively.

Promisingly, systematic studies on the aqueous solubility of degraders are currently emerging in the literature.^72,73^ To directly measure the thermodynamic aqueous solubility of a substance, Log S, traditional saturation methods (in combination with LC/MS analysis) or potentiometric methods are often applied.^74^ In particular, for degraders that contain ionizable sites, the pH-dependent solubility can be assessed and the intrinsic solubility, Log S_0_, i.e., that of the neutral species, reported.^75^ Also, for poorly soluble compounds like many degraders, dissolution profiles from amorphous solid or liquisolid formulations are sometimes derived.^76^

Since different degrader chemotypes can penetrate different cell types, it has long been argued that their cellular uptake occurs through passive diffusion. Recently, common label-free methods that measure the cell permeability, such as the parallel artificial membrane permeability assay (PAMPA)^77,78^ or the Caco-2 cell monolayer assay,^79^ have shed light on degrader permeabilities.^80,81^ To increase the sensitivity, a HaloTag-based assay, called the chloroalkane penetration assay (CAPA), or even system-specific intracellular reporter-based assays have been designed that helped rank degraders based on their permeability.^82,83^ In an interesting approach, Atilaw et al.^84^ applied NMR spectroscopy to study degraders with different physicochemical properties in polar and nonpolar solutions, thus providing a structural basis for their permeability differential. The recent development of new technologies and the creative use of existing tools for the analysis of both the solubility and permeability of degraders will advance our knowledge of distinct features that govern their bioavailability.

As mentioned earlier, once degraders reach their targets, their mode of action is different from that of inhibitors. In particular, degraders induce two binding events. The corresponding binding kinetics are usually assessed by surface–plasmon resonance (SPR) that informs on the lifetime or stability of the degrader-induced ternary complex and the associated (un)binding rate constants.^37^ Since the protein is typically attached to a surface through a tag, the native binding properties of the target protein may be disrupted in SPR. Nevertheless, these measurements are instrumental in characterizing a degrader’s binding affinity to both the POI and the ligase and hence its degree of binding cooperativity, i.e., the increase in a degrader’s affinity to either binding partner in a ternary complex compared to the corresponding affinity in a binary (nonternary) structure. Cooperativity is a key parameter to determine degrader specificity.^38^

Competitive-binding and proximity-based assays can also probe the degree of degrader–POI engagement or ternary complex formation (Table 1), thus allowing the determination of binding affinities and cooperativity. These techniques measure changes in the polarization of a fluorescently labeled target protein upon binding to a degrader, as in fluorescence polarization (FP),^85^ or detect intensity changes, upon ternary complex formation, in the fluorescence or luminescence signal emitted from beads tagged to the POI or E3 ligase, as in time-resolved Förster resonance energy-transfer (TR-FRET)^86^ or in the amplified luminescent proximity homogeneous assay (ALPHA).^87^ In the proximity ligation assay (PLA), a technology that is gaining popularity in TPD research, the signal is even further amplified through antibody-attached oligonucleotide hybridization.^88^ These methods have been used in TPD studies for the identification of selective degraders and for the high-throughput screening of degrader designs.^39−41,89^ In particular, ALPHA has a high dynamic range and signal-to-noise ratio and, when applied in a titration experiment (usually set up as an immunoassay called AlphaLISA), it yields relative populations of ternary complexes as a function of degrader concentration and is routinely employed as a tool in degrader design projects (see Section 4.2).

Complementary to the methods described above, isothermal titration calorimetry (ITC) is a label-free technique and thus does not require tagging or immobilization of the analytes. ITC measures the heat released or absorbed during the binding of a degrader to its target protein, providing information on binding affinity, stoichiometry, and the changes in enthalpy and entropy, although at lower throughput and higher protein demands. The ITC-guided optimization of degraders has been reported for different POI–ligase pairs, e.g., Tau-KEAP1^42^ and BRD4-VHL.^40^

Alternatively, NMR spectroscopy, which is based on chemical shift perturbations upon ligand binding, is fairly sensitive to binding signals^90^ and has been used for fragment screening on protein surfaces,^91,92^ in particular to identify binding pockets on the von Hippel–Lindau (VHL) E3 ligase^93^ and to aid in the optimization of its inhibitors.^94^ Recently, Castro and Ciulli reported an NMR assay that facilitates the evaluation of cooperativity in degrader-induced ternary complexes.^95^

X-ray crystallography has been used to resolve the structures of entire ternary complexes,^96^ or those of bound warhead–POI^44^ or E3 ligand–ligase,^97^ revealing distinct interactions in atomic detail and providing invaluable structural knowledge for rational degrader design. However, X-ray crystallography is a very challenging technique and hence cannot always be applied, particularly not for relatively large and, yet, dynamic ternary complexes that are difficult to co-crystallize. This issue can be remedied by cryogenic electron microscopy (cryo-EM) that solves large macromolecular structures, including ternary complexes.^98^ Cryo-EM is generally considered to become an essential tool in drug discovery,^99^ and we believe that it will play an important role in the discovery of novel degraders.

Other (label-free) tools that have been used to provide insight into degrader–protein engagement and ternary complex formation or ubiquitination include size-exclusion chromatography (SEC), that can compare the degree of complex formation among different degraders,^100^ native mass spectrometry (MS), that has helped explore degrader selectivity and specificity,^101^ and differential scanning calorimetry, that, when applied as a cellular thermal shift assay (CETSA),^102^ exploits the differential in the thermal stabilization upon ternary aggregation in a cellular environment.^38^

The Western blot immunoassay, after separation of protein samples by gel electrophoresis, is frequently used to measure the target protein levels inside the cell, thus assessing the degrader activity. Although capillary electrophoretic immunoassays are believed to be less error-prone,^103^ Western blot, coupled with other assays, has become a standard tool to probe target protein ubiquitination and degradation.^28,37−47^

Both fast photochemical oxidation of proteins (FPOP) and hydrogen–deuterium exchange (HDX) are MS-based footprinting methods that report on the solvent accessibility of proteins, which is altered upon conformational changes, ligand binding, or consequently, ternary complex formation. These two methods characterize the protein binding interface in a fairly complementary fashion,^104^ as different regions are labeled (side chains versus backbone) and, as a result, different time scales can be assessed (ns−μs versus ms–s).^105,106^ In particular, HDX-MS has already been applied to elucidate protein sites that contribute to ternary complex formation, and the information provided can easily augment follow-up modeling and simulation analyses.^107,108^ In our opinion, these structural MS techniques can be ideally supported by other chemical footprinting or even site-directed mutagenesis experiments in which the role of distinct residues at the binding interface can be intensely explored.

Mass spectrometry techniques are pivotal for quantitative proteomics to characterize protein modifications and thus assess target ubiquitination and degradation.^109,110^ For instance, when coupled to *in vitro* ubiquitination assays, MS analysis has identified distinct degrader-induced ubiquitination sites and (poly-)ubiquitination linkage types,^40,111,112^ although the coverage of ubiquitinated lysines may be incomplete in these experiments. Recent advances in live-cell ubiquitinomics involve the transfection of HeLa cells with the tagged POI and ubiquitin, followed by immunoprecipitated ubiquitin pull-down and electrophoretic or LC/MS analysis.^38,108^ MS-based global proteome profiling can quantitate the abundance of target proteins, i.e., their turnover and resynthesis rates, and therefore the effective degrader response^28,39,44^ and possibly identify off-target effects.^113^ Proteomics approaches are believed to play an outstanding role in TPD research.^114^

To monitor the intracellular degradation, luminescence-based reporter assays with endogeneous HiBiT-tagged proteins have been developed that yield degradation profiles, from which maximum degradation levels (*D*_max_) as well as the half-maximum degradation concentration (DC_50_) can be derived to estimate the potency of a degrader molecule,^48^ which is instrumental in degrader design (see Section 4.2). As a matter of fact, variants of this technology are applied to characterize the different steps in TPD, such as target engagement, ternary complex formation, and target ubiquitination,^115^ providing complementary kinetic information to the other experimental approaches discussed.

To evaluate degrader activity phenotypically, the target phenotype is often compared to those from CRISPR/Cas9-mediated protein knockouts or from RNAi screens.^48,49^ Recent techniques even employ reporter genes to reflect the expression level of target proteins. Measuring the intracellular downstream effect of TPD, such as changes in cell viability,^51^ proliferation,^50^ or morphology,^52^ is a functional readout that can provide insights into the biological consequences of degrading the target protein.

The variety of methods being deployed in TPD projects attests to the complexity of the TPD process and mirrors the fact that multiple molecular events must take place in order to achieve degradation. In combination with molecular modeling, this large suite of technologies has already helped advance our knowledge and understanding of the different TPD steps for distinct POI–ligase pairs. For the sake of completeness, we would like to note that multiple other approaches, not listed above, have also been recently applied.^116^ Furthermore, the current toolbox is rapidly expanding as new innovative methods are being developed to provide greater detail about the TPD-related processes.

## 3 Modeling the TPD Process

**Figure 3:**
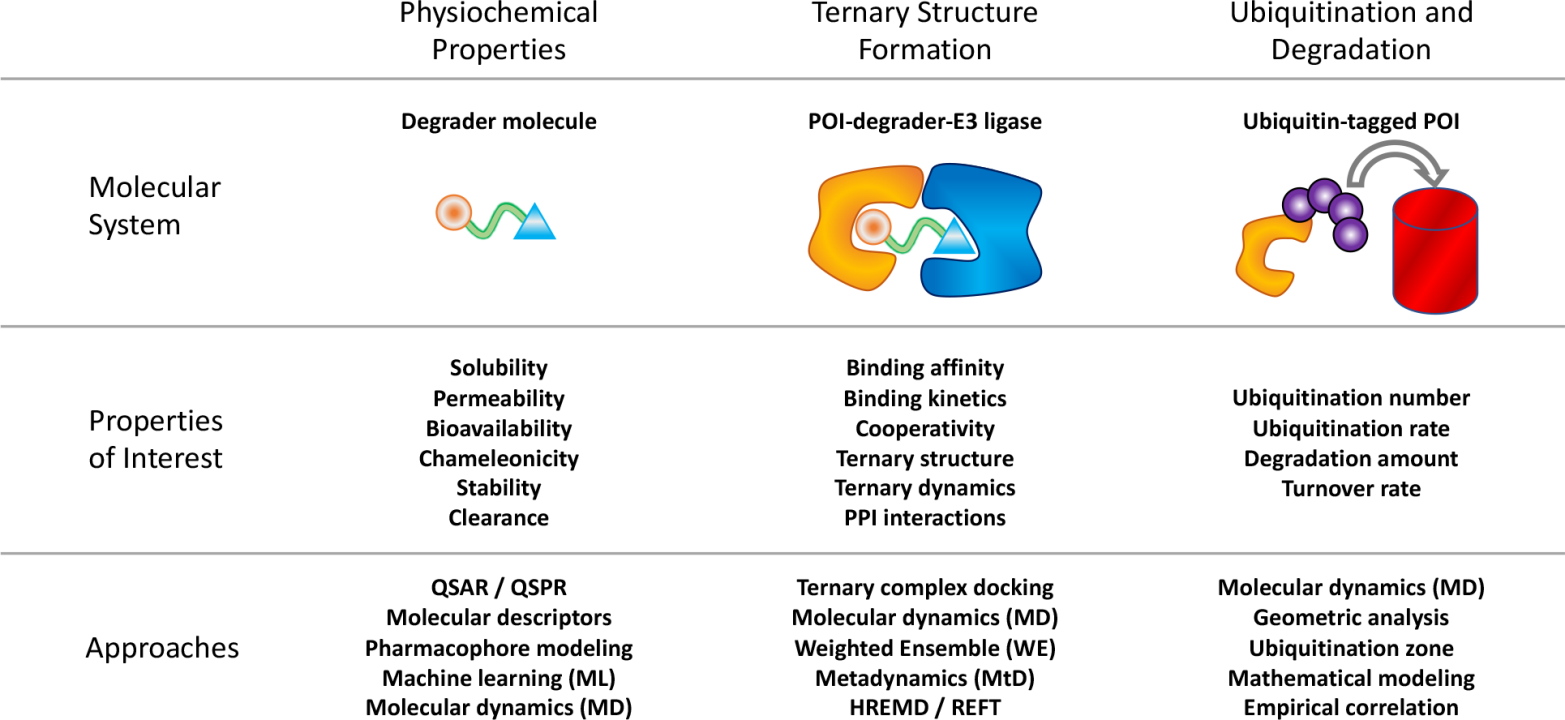
Properties of interest and computational approaches in TPD. The multistep process of TPD entails a diverse set of modeling techniques developed for the prediction of degrader physicochemical properties, degrader-induced ternary structures, and the degree of target ubiquitination and degradation. Higher-level properties such as pharmacokinetics (PK) and pharmacodynamics (PD) could encompass all of these properties, plus additional properties (e.g., tissue localization of the degrader, metabolites, and off-target effects). This figure is intended to be suggestive and not exhaustive. The approaches described here are software-agnostic and could be performed with a variety of open-source and commercial software packages.

Despite the breadth of biochemical and biophysical tools developed and adapted for exploring TPD, an integrated approach to model the different steps can lead to more holistic predictions of the entire degradation process.^117^Figure 3 shows a summary of properties that help characterize different stages of the TPD process and the state-of-the-art modeling techniques applied for their study.

While pharmacokinetic–pharmacodynamic (PK–PD) and other quantitative models formulate the multistep TPD mechanism mathematically, it is possible to model some aspects of the process on a molecular level using quantitative structure–activity/structure–property relationships (QSAR/QSPR)^118^ as well as molecular modeling and simulation.

In QSAR/QSPR, multivariate regression and classification analyses, augmented by a variety of machine learning (ML) methods,^119−122^ are applied to predict physicochemical traits, that determine ADMET-related effects, based on a set of molecular descriptors, typically representing the chemical structure of a compound at the level of its structural formula (2D) or its conformation (3D).^123^ As a result of increased training data availability and advances in deep learning methodologies, graph convolution models that learn a molecule’s structural representation have recently been developed for property prediction.^124−126^ However, as noted earlier, for most “beyond rule of 5” (bRo5) compounds and particularly for heterobifunctional degraders, the accurate prediction of physicochemical properties is quite challenging. Recently, some approaches have been put forward to address this issue.^127−130^

The prediction of degrader properties can be enhanced by molecular dynamics (MD) simulations, which have become an important tool in computation-enabled drug discovery assisting in the identification of ligand binding sites on protein surfaces,^131−133^ augmenting docking routines,^134−136^ and predicting protein–ligand binding affinities.^137−139^ Compared to current QSAR methods, which often exploit topological descriptors, simulation trajectories are information-rich, ideally containing a full mechanistic and dynamic description of the system, providing useful information on the thermodynamics and kinetics of a drug–target binding process^140^ in addition to extracting 3D descriptors, such as the radius of gyration or intramolecular hydrogen bonding. We elaborate in the following paragraphs why we expect MD simulations to be integral in many aspects of TPD modeling and degrader design.

### 3.1 The Prediction of Degrader Physicochemical Properties Is Challenging

Traditionally, the computational assessment of a drug’s thermodynamic aqueous solubility ranges from predictions of properties such as melting points (T_m_)^141^ or partition coefficients^142^ and the application of QSPR methods^143,144^ to the estimation of solvation free energies^145−147^ and solubility parameters from molecular simulations.^148−150^ In recent years, ML methods for the prediction of solubilities in water and in organic solvents have become popular.^151−153^ Recent applications of predictive models to degrader molecules, or similar high-molecular-weight compounds in the bRo5 space, suggest that the same rules developed for small molecules cannot be readily transferred.^154,155^ In particular, the structural flexibility of large heterobifunctional degrader molecules requires model revisions such that certain parameters that support chameleonicity (the ability of a molecule to adopt both hydrophobic and hydrophilic 3D conformations) are combined with those that favor oral bioavailability (e.g., the distribution coefficient, Log D).^156,157^ Moreover, the dynamic nature of features, such as the changing exposure of surface polarity, seems to better capture the solubility (and permeability) of bRo5 compounds than traditional two-dimensional descriptors do.^29^

To systematically distinguish degraders from nondegraders in their solubility, Jiménez et al.^72^ trained a decision tree classifier that relies on correlations between a set of experimentally obtained lipophilicity descriptors and several *in silico* solubility predictors based on the structure of ∼20 heterobifunctional molecules. Furthermore, they also examined the effect each of the three different motifs within a heterobifunctional molecule, i.e., the warhead, the linker, and the E3 ligand, could have on a degrader’s solubility. Despite the fact that they derive preliminary guidelines to identify soluble degraders, this study reveals, as the authors admit, the complexity of the task of designing orally bioavailable degraders.

Similar to the prediction of solubility, QSPR methods have also been established between results from experimental permeability screens, e.g., PAMPA, and structural descriptors such as the intramolecular hydrogen bonding,^158^ solvent-accessibility,^159^ or molecular size of the permeant.^160^ Often, these models use an expression for the water–membrane partitioning^160^ or free energy of transfer.^161^ To sample sufficient conformations, MD simulations are routinely applied^162,163^ and, in particular for bRo5 compounds such as macrocyclic^164,165^ or degrader molecules,^166^ conformational sampling has assisted in predicting permeability.

Over the past decade, reports on atomistic simulations of the entire process of passive membrane transport have multiplied in the literature, as previously reviewed.^167−169^ For drug molecules of different sizes, experimental permeability coefficients have been estimated based on the solubility–diffusion model^170−174^ and, notably, distinct permeation pathways of (small-molecule) compounds have even been detected,^175^ highlighting the potential of novel simulation algorithms, in combination with more realistic models of cell membranes,^176^ to advance our understanding of the cellular uptake of drugs. Yet, despite these successes, all-atom simulations of translocation across membranes remain costly to be applied at scale for relatively large molecules like degraders. To this end, low-dielectric continuum or implicit membrane models^177−181^ and (physics-based) mechanistic predictors, such as PerMM,^182,183^ COSMOperm,^184^ or other tools,^185^ have been developed, which allow higher throughput, albeit at the cost of accuracy.

The application of ML methods for the prediction of membrane permeability has also sharply increased in recent years. Some examples include neural networks, support-vector machines, and random forest classifiers trained on molecular structures and physicochemical properties of small and large compounds.^186−189^ To predict degrader permeability, Poongavanam et al.^190^ have tested several binary classifiers using descriptors that represent molecular size, shape, and chemical functionalities. While predictions were good (accuracy >80%) in some cases (e.g., VHL-recruiting degraders), the classifiers performed poorly on cereblon-recruiting degraders due to imbalances in the corresponding training data. This emphasizes the need for more high-quality degrader data sets, especially given the hundreds of E3 ligases in the human genome, many of which have minimal TPD data. Rather than directly measuring permeability, which tends to be challenging for heterobifunctional degrader molecules, cellular target engagement assays, described in more detail in Section 2, are often deployed to assess whether a molecule gets into the cell or not (although permeability is not measured directly through this approach).

The examples presented above, as well as other references in the literature,^66,154^ highlight the two obvious difficulties in the prediction of degrader properties such as solubility and permeability. First, due to their relatively large and heterobifunctional structure, degraders can adopt a multitude of different conformations that, unlike small-molecule drugs, can lead to very different molecular interactions, thus complicating traditional property-based drug design. Yet, it is this structural flexibility that leads to the aforementioned degrader chameleonicity, which allows both solubility *and* permeability. Conformational sampling via molecular dynamics, Monte Carlo, or other techniques can be used to capture these effects and generate molecular descriptors of degraders, which we believe to be important in degrader design. Molecular simulations can furnish conformational ensembles that help to derive structural attributes, such as hydrogen bonding and solvent accessibility, in different environments.^191,192^ The use of such 3D descriptors, in particular for the prediction of solubility and permeability, is currently an active area of research.^29,164,165,193,194^

The second main issue in TPD modeling is that accurate prediction of degrader properties is currently strongly hampered by the lack of experimental data. Although it had been argued before that the actual QSPR algorithms, rather than the uncertainty in data measurements, may be responsible for inaccurate solubility predictions,^195^ it is universally accepted that limitations on training data impair prediction accuracy, especially for ML-based approaches. Robust predictive modeling of physicochemical traits, such as solubility and permeability, requires a large and representative set of curated data,^196^ which are scarce for degrader molecules. In this context, the ∼2-fold expansion of PROTAC-DB 2.0,^197^ one of the primary repositories on structural and experimental data on degraders, which has recently grown to over 3,200 entries at the time of this writing, mitigates this dearth of knowledge to some extent. However, the data are still sparse given the complexity of the problem. We expect that the collection of information about degraders, including their chemical structures, biological activities, and physicochemical properties, will continue to grow over the next decade. To compensate the lack of available experimental data in the near future, data resampling techniques^198,199^ may be applied that, in combination with dynamic structural features readily available through molecular simulations, could facilitate the development of models for degrader classification.

### 3.2 Conformational Sampling of Degrader-Induced Complexes Plays an Important Role in TPD Modeling

The most-studied step in the TPD process by means of molecular modeling is the degradation-induced formation and conformational sampling of ternary complexes. As discussed, structure-based biophysical experiments, in particular, X-ray crystallography, have previously helped in the rational design of new degraders. However, since ensembles of structures better aid in understanding structure–function relationships than static structures do, the optimization process of potent degraders can strongly benefit from accurate modeling of ternary structures, i.e., the prediction of distinct molecular interactions that promote complex formation, thus characterizing the underlying selectivity and cooperativity.

For a robust assessment of predicted ternary complexes, the structural flexibility of degraders along with the variability in productive POI–ligase poses requires a comprehensive sampling of possible conformations of all components. For example, it has been shown that the linker motif impacts the ternary complex formation and degradation efficiency.^200,201^ Furthermore, the notion of cooperativity calls for a representative set of protein–protein assemblies to identify possible degrader effects. To this end, several prominent docking programs, often augmented with MD, have recently been employed for the design of selective degraders.^38,45,89,202,203^

Ternary complex docking has emerged as an extension of traditional docking methods. Molecular docking is a standard tool for pose prediction and virtual screening in drug discovery.^204,205^ In the context of degrader-induced TPD, both protein–ligand^206^ and protein–protein^207^ docking may be applied to generate structures that are not experimentally resolved. In fact, several protocols have been developed that differ by the order in which degrader conformations are sampled, i.e., either after all components are docked into a ternary complex, or only in a binary protein–degrader complex before superposing the second protein, or even before inserting the degrader with its sampled conformation into a given protein–protein aggregate.^208^ While each docking protocol attempts to minimize the number of predicted structures with sterically clashing components, those sampling the POI–ligase interactions and the degrader conformations separately before combining all parts into a ternary complex have become most popular due to their higher accuracy in recapitulating experimentally known or crystal-like structures.^209−211^ Typically, repurposing recently developed automated routines,^212−214^ a library of linker conformations is combined with the warhead and the E3 ligand motifs, that are possibly docked to structures from the POI and the E3 ligase, thus generating ternary complex models, which are scored by an energy function to identify favorable combinations.^28,40,45,89^ The interactions within each protein are unlikely to change significantly in the context of the ternary complex, simplifying the search for high-scoring models. Although recent work argues for the simultaneous docking of all three components for higher accuracy,^215^ in some instances, the binding modes of the warhead to the target POI and that of the E3 ligand to the E3 ligase are known, allowing for constraints to be applied to the ternary complex docking problem. Specifically, the use of such structural information on the ternary complex interface obtained from HDX experiments, as implied above, has been shown to significantly boost the performance of ternary docking protocols.^107,108^

Considering the relatively intricate problem of ternary complex formation, MD simulations hold great promise for rationalizing structure–activity relationships among the different binding partners. To date, simulations of ternary complexes have been applied for tasks such as analyzing interactions between the three binding partners in atomistic detail,^216^ probing the binding cooperativity,^217^ scoring and ranking given degraders,^218^ and assessing the stability of degrader variants, such as covalently binding degraders,^219^ macrocyclic degraders,^43^ or such aimed at new diseases.^220^ In particular, molecular simulations have successfully augmented many biochemical and proteomics assays to inform on the selectivity of distinct degraders^38,89,221,222^ and also structural biophysical techniques to elucidate binding site interactions.^40,93,223^ Recently, our research team has combined MD simulations with X-ray crystallography, small-angle scattering, HDX, and ubiquitinomics experiments to explore the differential in DC_50_ values between three VHL-recruiting SMARCA2 degraders.^108^ This rather comprehensive work provides an explanation for degradation efficiencies as primarily influenced by the stability and geometry of ternary complexes, particularly in the context of the entire Cullin–RING ligase (CRL). Based on simulations, we found that productive ternary complexes, i.e., such with a high ubiquitination probability, do not coincide with the crystal structures, which presumably are dominated by crystal contacts, but rather with the global energy minima, thus demonstrating the value of advanced simulations for the study of the TPD process.

**Figure 4:**
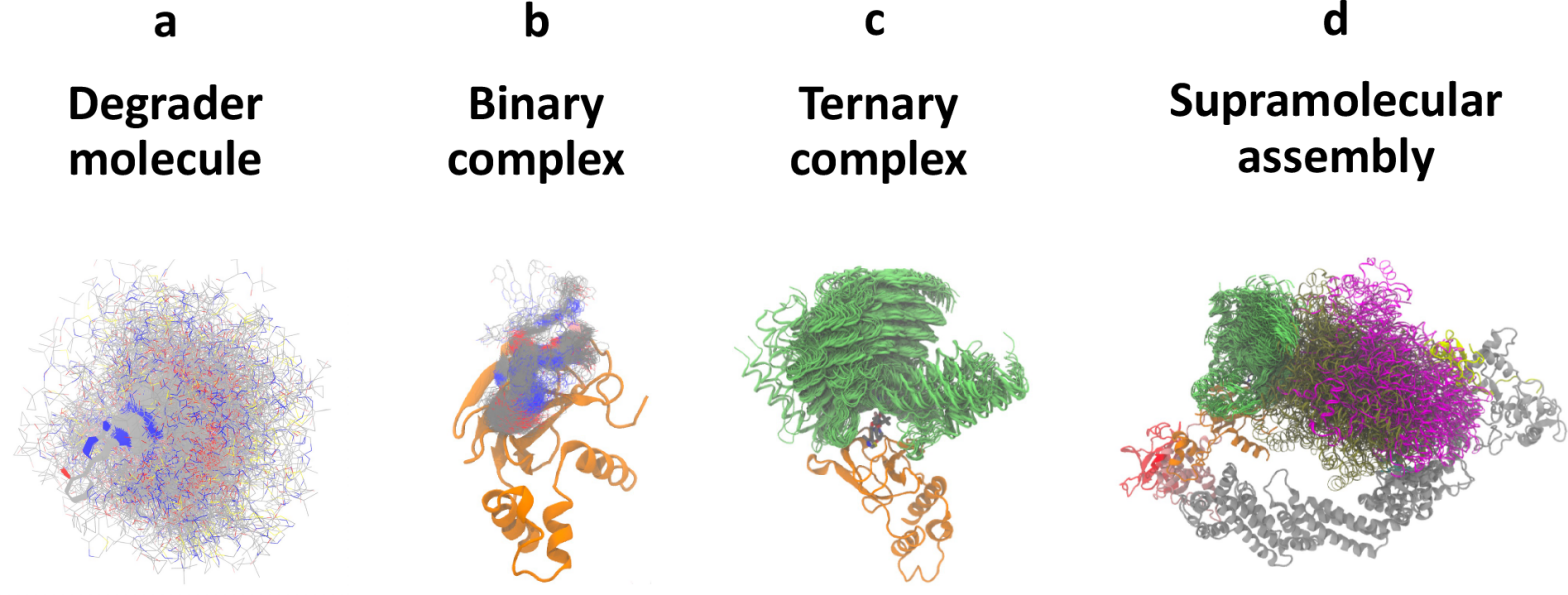
Conformational sampling from MD simulations of (a) a degrader molecule in solution, (b) a degrader–ligase binary complex, (c) a POI–degrader–ligase ternary complex, and (d) a POI–degrader–CRL supramolecular assembly (not to scale). As explained in the main text, simulations can be used to predict the properties of degraders, the affinity of binary complexes, dynamic ensembles of ternary complexes, and the likelihood of ubiquitination in the context of the CRL.

In our opinion, MD simulations should be fully integrated in degrader design cycles (see Section 4) as a means to provide mechanistic information as well as thermodynamic and kinetic parameters for the decision-making process. Figure 4 displays the structural variability of the degrader molecules and distinct degrader-induced complexes sampled by MD simulations. Despite the structural complexity of the molecular systems and the associated time scales of the processes involved in ternary complex formation and target ubiquitination, modern enhanced sampling algorithms, in combination with graphical processing units (GPUs), offer substantial progress in the size and time scale of simulations that can be routinely performed.

Specifically, simulations can augment and greatly improve the accuracy of ternary complex docking protocols, more so than already acknowledged in the context of small-molecule docking.^224,225^ MD applied to docked ternary complexes can assess their quality and possibly simulate transient and (meta)stable states. In contrast, applying ternary complex docking *after* the simulation of adjacent POI–ligase pairs that lack a degrader, so-called “encounter complexes”, allows the assembly of a ternary complex with distinct protein–protein interactions, which we find a very appealing strategy. From a statistical–mechanics viewpoint, the presence of a heterobifunctional degrader between two proteins corresponds to a constraint leading to sampling of a confined subspace within the full encounter complex configurational space. Therefore, simulations of degrader-less encounter complexes exhibit the baseline interactions of a given POI–ligase pair, which are not accessible from simulations of the full ternary complex, providing knowledge that can be exploited for rational degrader design.

Path-sampling strategies are employed to simulate nonequilibrium phenomena, such as the degrader-induced assembly of proteins. These methods, also referred to as importance-sampling techniques, usually require a predefined collective variable along which phase space is sampled, accomplished by the addition of biasing potentials as in umbrella sampling^226^ or steered or accelerated MD^227,228^ or similar methods, that apply the potentials in an adaptive fashion.^229−231^ One such method, that has become popular in computational drug discovery, is metadynamics^232−234^ and its many variants that converge faster,^235−239^ which, in our study, were adequate for the simulation of large structural changes in the RING-finger E3 multiprotein ligase complex connected to SMARCA2 by a degrader.^108^ Simulations of large conformational changes of the ubiquitination system are instrumental for modeling the TPD process and, in our opinion, enhanced sampling methods are key in this regard. For instance, simulations that mechanistically describe the ubiquitination process can model the presentation of the target substrate, i.e., its proximity and orientation, with respect to the ubiquitin-carrying enzyme, thus supporting the assessment of degrader efficacies or the degree of (poly)ubiquitination of distinct sites.

In contrast to the methods described above, rigorous path-sampling schemes introduce less bias into the dynamics and thus do not require any additional assumptions for the calculation of rate constants of the simulated rare event. These include, among other methods, transition path sampling^240,241^ or dynamic importance sampling,^242^ in which complete transition paths are iteratively refined or reweighted, or strategies that construct paths by simulating many short trajectory segments, such as forward flux sampling,^243^ milestoning,^244^ weighted ensemble,^245,246^ or innovative combinations thereof.^247^ In the context of ternary complex formation and ubiquitination, we consider these simulation techniques to be essential, as they yield kinetic and thermodynamic information on binding events, thus complementing experiments like SPR and ITC. Importantly, the simulation of multiple (weighted) binding pathways can reveal the impact of degrader molecules on protein–protein association. By estimating transition rates, these simulations can reveal which pose of a ternary complex is most favored and thus measure the likelihood of distinct sites to be ubiquitinated. In fact, we have shown the capacity of weighted ensemble simulations to predict ternary complex binding pathways and reproduce binding rate constants that are in line with experiments.^108^ Notably, the simulations gave more accurate results, particularly in comparison to ternary complex docking, when the collective variable included information from HDX experiments using an affordable amount of resources.

Generalized ensemble techniques, such as parallel tempering,^248,249^ are orthogonal to path sampling in that the simulation is enhanced in a path-independent manner, for instance by raising the temperature. We have shown that such an approach is beneficial for the exploration of ternary complex structures and dynamics, producing an accurate description of conformational states upon projection onto a free energy surface that aids in identifying metastable states within the dynamic ternary system.^108^ Specifically, solute scaling (or Hamiltonian replica-exchange MD, HREMD)^250^ and flexible tempering (REFT),^251^ in which the Hamiltonian of the whole solute or only parts of it are scaled, are promising alternatives to sample the structures of ternary aggregates as they are more efficient than the traditional temperature REMD. A further option to efficiently explore the free energy landscape of ternary complexes is the coupling of REMD to a reservoir of different conformations,^252,253^ that could stem from ternary complex docking, virtually leading to the simulated annealing of a docked ensemble.

The methods described can simulate degrader binding, ternary complex formation, and conformational changes (Figure 4). Still, considering the size and the flexibility of the ternary systems, sophisticated analytical treatments need to be combined with the enhanced simulations, possibly even as iterative reweighting schemes.^254,255^ These approaches often project the dynamical evolution of the system in space and time onto a model, as in master equation representations,^256^ capturing the conformational dynamics of the system and enabling long-time scale predictions. A popular concept is the use of Markov State Models (MSMs), which, over the past decade, have been applied to a large number of biomolecular systems.^257−260^ MSMs provide one of the best ways of coarse-graining the dynamics of the ternary complex ensemble and identifying high-probability conformational states that can be correlated to ubiquitination scores and other properties. This strategy can help identify specific residues that may be of particular importance to transitions among different states and also quantify the effect of degraders on the dynamics of the ternary system.

Finally, we should mention the recent rise in *de novo* protein structure prediction, based on contacts and sequence information from known structures,^261−265^ as an avenue to predict conformations of productive ternary complexes. While these approaches are promising, their application to larger protein aggregates, such as ternary complexes, is more complicated and has not been reported yet. Moreover, in contrast to many simulation methods, they lack information about the assembly and structural variability of ternary complexes. Nevertheless, we consider that the generative modeling of degrader-induced ternary complexes will become an important analysis tool for TPD modeling as more knowledge on the determinants of productive structures is being accumulated.

The methods outlined above highlight the significance of advanced molecular simulations, not only in understanding the fundamentals of TPD but also as a routine application tool for degrader design. As we describe in more detail in Section 4, computation-enabled degrader design relies on the orchestration of automated procedures to efficiently scan individual degrader candidates. The simulation approaches presented here provide a set of tools that are very well-suited for this task.

### 3.3 Mathematical Models Can Help Determine Properties to Optimize Degradation

There is a complex relationship within the *in cellulo* and *in vivo* environments between the aforementioned steps in the TPD process. Complementary to molecular modeling, mathematical frameworks furnish a representation of the TPD process at larger time and length scales. In addition to the relationships across solubility, permeability, binding, ubiquitination, and degradation, there are subtleties associated with the stability of the ternary complex, which is influenced by the interactions between the degrader molecule, the POI, and the E3 ligase. The resulting positive or negative binding cooperativity during ternary complex formation can influence the overall degradation efficiency. However, the impact of cooperativity on degradation, mediated by a heterobifunctional molecule, remains an open question. In a recent study by some of the authors of this Perspective, a pharmacodynamic model was developed to describe the kinetics in the TPD process and it was used to explore the role of cooperativity in ternary complex formation and POI degradation.^266^ The model established a quantitative relationship between the stability of the ternary complex and degradation efficiency by examining the effect of the complex stability on the rate of catalytic turnover. Additionally, the authors devised a statistical inference model to determine cooperativity in intracellular ternary complex formation using cellular assay data. The work was validated by quantifying changes in cooperativity due to site-directed mutagenesis at the POI–ligase interface of the SMARCA2-ACBI1-VHL ternary complex. This pharmacodynamic model is an example of a quantitative framework to dissect the intricate degrader-mediated TPD process, which could inform the rational design of effective heterobifunctional degraders. By contextualizing these findings with the experimental techniques described above, it should be possible to provide a more comprehensive understanding of the factors influencing the success of TPD-based drug discovery and therefore a more rational approach to optimizing degrader properties for TPD.

In a related work, researchers developed an extensive modeling framework to analyze experimental data for three primary objectives: (1) evaluate degraders using precise degradation metrics, (2) optimize crucial compound parameters, and (3) link degradation to subsequent pharmacodynamic effects.^267^ The proposed framework introduces several novel features: (1) a mechanistic model to fit the hook effect observed in degrader concentration–degradation profiles, (2) quantification of the role of target occupancy in the mechanism of action, and (3) disentangling the effects of target degradation and inhibition on the overall pharmacodynamic response. The authors demonstrate the applicability of this approach by applying these three models to analyze exemplary data from multiple compounds, projects, and targets. The framework enables researchers to tailor their experimental work, leading to a deeper understanding of their results and ultimately facilitating a more successful degrader discovery. Although the focus of this work was on *in vitro* pharmacology experiments, the key implications for *in vivo* studies are also discussed.

Along the same lines, a general model for ternary complex catalysis has been developed within a framework familiar to classical enzyme theory.^268^ The authors adapted a strategy within Michaelis and Menten’s original publication (integration of the velocity equation) to solve for the maximum velocity (*V*_max_).^269^ These equations are straightforward to implement and enable estimation of time scales that are consistent with a wide range of published literature values. Additionally, the authors validated these equations with thermodynamic and kinetic databases and built an interactive web tool that enables researchers to graphically simulate their own systems. Other reviews have discussed the current state and future directions of TPD drug discovery in the context of building a quantitative relationship between loss of protein target and *in vivo* activity,^270^ where mechanistic PK–PD models are highlighted with the aim to improve the translation from the preclinical to clinical space.

## 4 Computation-Enabled Degrader Design

Early in degrader discovery programs, when experimental information is sparse, computational modeling can be leveraged to design and prioritize molecules for synthesis. The process of designing degraders includes multiple complex steps incorporating computational tools, which have the advantage of prospectively generating degraders and computing quantitative rankings that can inform decision-making in discovery projects. To this end, we have developed a workflow that comprises the modeling and simulation methods presented above to guide the degrader design and assess the suitability of degrader candidates.

### 4.1 A Design Strategy Based on the Integration of Computational Tools

While both the warhead and the E3 ligand motifs within a heterobifunctional degrader molecule are essential for ternary complex induction, a multitude of studies have demonstrated that potent and efficacious degradation of a target POI is also dependent upon the conjugation vector and, as noted above, the chemical structure of the connecting linker motif.^89,271,272^ In this vein, many computational tools for degrader design focus on linker conjugation and optimization. Biophysical and structural studies have revealed critical insights into how linkers influence the positioning of the POI in relation to the ligase, either positively or negatively regulating its ubiquitination.^28,45^ These studies and biochemical investigations alike have also shown how alterations in chemical linkers can facilitate cooperativity and stability of ternary complexes, often leading to improved and more sustained degradation.^28,37,40^ Structural studies and the advancement of molecular modeling of larger protein complexes have guided degrader linker design for targets and ligases with prior information available.^23,273,274^ Therefore, combinatorial approaches for degrader design typically use a small number of warheads and E3 ligands and consider their associated attachment points and linker libraries. A good source of linkers is the patent literature on degraders, commercially available linker libraries, and the aforementioned publicly available PROTAC-DB.^197^

A good degrader must be synthetically feasible, chemically stable, and bioavailable. Thus, similar to small-molecule drug design cycles, any computational prediction on degrader properties should be followed by decision making in collaboration with synthetic and medicinal chemists. Considering the many different types of linker motifs recently suggested in the literature (for a good overview, see Figure 1 by Desantis et al.^275^), expert opinion is imperative for degrader design. For instance, synthetic accessibility constraints should be employed, and synthetic handles defined *a priori* to ensure computational predictions can be validated experimentally.

An important step in degrader design is certainly the use of physicochemical and ADMET property predictions to filter the number of compounds being considered. However, as discussed above, many of these methods are currently being improved for degraders and will play a more significant role in the near future. For the prediction of permeability, we found that running MD simulations for 0.5 μs to obtain ensembles of structures and thus distributions of quantities, such as the linker end-to-end distance, provides more accurate results than applying predictors to a single structure. Also, based on our experience, the prediction of Log P values with (physics-based) mechanistic tools, such as PerMM,^182,183^ is suitable to provide a correct trend among multiple degraders.

The fastest way to obtain a ternary structure of a given POI–ligase pair connected by a degrader candidate molecule is via ternary complex docking. As we described earlier, the actual protein conformations are not changing significantly during this procedure; therefore, the key challenge in ternary complex docking entails computing the orientation of the two proteins relative to each other. We use ternary complex docking with two major modifications. First, rather than using rigid protein–protein orientations generated by protein–protein docking, we use conformations coming from MD simulations of the encounter complexes (augmented by Markov State Modeling, as described below) as the starting point for docking the degrader candidate to form a ternary complex, thus, capturing important baseline interactions between the POI and the E3 ligase in order to achieve accurate ternary models. Second, those ternary complexes that minimize clashes, preserve warhead and E3 ligand binding modes, and, if known, look similar to ternary complexes of active degraders are scored highly, whereas those models that look similar to ternary complexes formed by known nondegraders are penalized. This orientational sampling thus defines what linker geometries are tolerated and preferred for each ternary complex.

Since a large number of different protein–protein poses or orientations may get sampled, the encounter complex simulations can produce a vast amount of data that require reduction. We apply Markov State Modeling (MSM) to the simulation trajectories and characterize their slowest-relaxing degrees of freedom with a Time–structure Independent Components Analysis (tICA),^276^ thus extracting a set of POI–ligase encounter complexes that, based on the given simulations, correspond to their metastable states. The significance of this simulation-driven approach is that degraders can be developed to optimize the balance between enthalpic and entropic contributions to ternary complex stability, for instance, such that they stabilize a given state with favorable interactions at the protein–protein interface or rather facilitate the conformational change between two metastable states. In our MSM protocol, we use K-means (or K-centers) clustering based on the number of contacts (i.e., heavy atoms within 5 Å) formed between the interprotein residues during the MD simulations. Importantly, despite the fact that the protein–protein simulation is more expensive than the rigid protein–protein docking, this simulation has to be performed only once for a given POI–ligase pair, resulting in a set of encounter complexes that can be reused for ternary complex docking of a myriad of degrader candidates.

We point out that the ensemble of simulated encounter complexes allows the direct design of degraders based on the baseline interactions mentioned above, which are, in practice, preferred protein–protein orientations or interfaces. This strategy essentially incorporates the design process into the ternary complex docking, virtually using the POI–ligase encounter structures as “templates” to draft a new degrader molecule.

Generating ternary complexes with candidate molecules should be followed up by MD simulations to examine their relative stability, preferably in the context of a supramolecular assembly to predict more accurately the ubiquitination probability of lysines in the POI. For estimating stability of the complexes, we recommend using proxy metrics for stability such as RMSD, RMSF, and the solvent-accessibility of lysine side chains over time.

**Figure 5:**
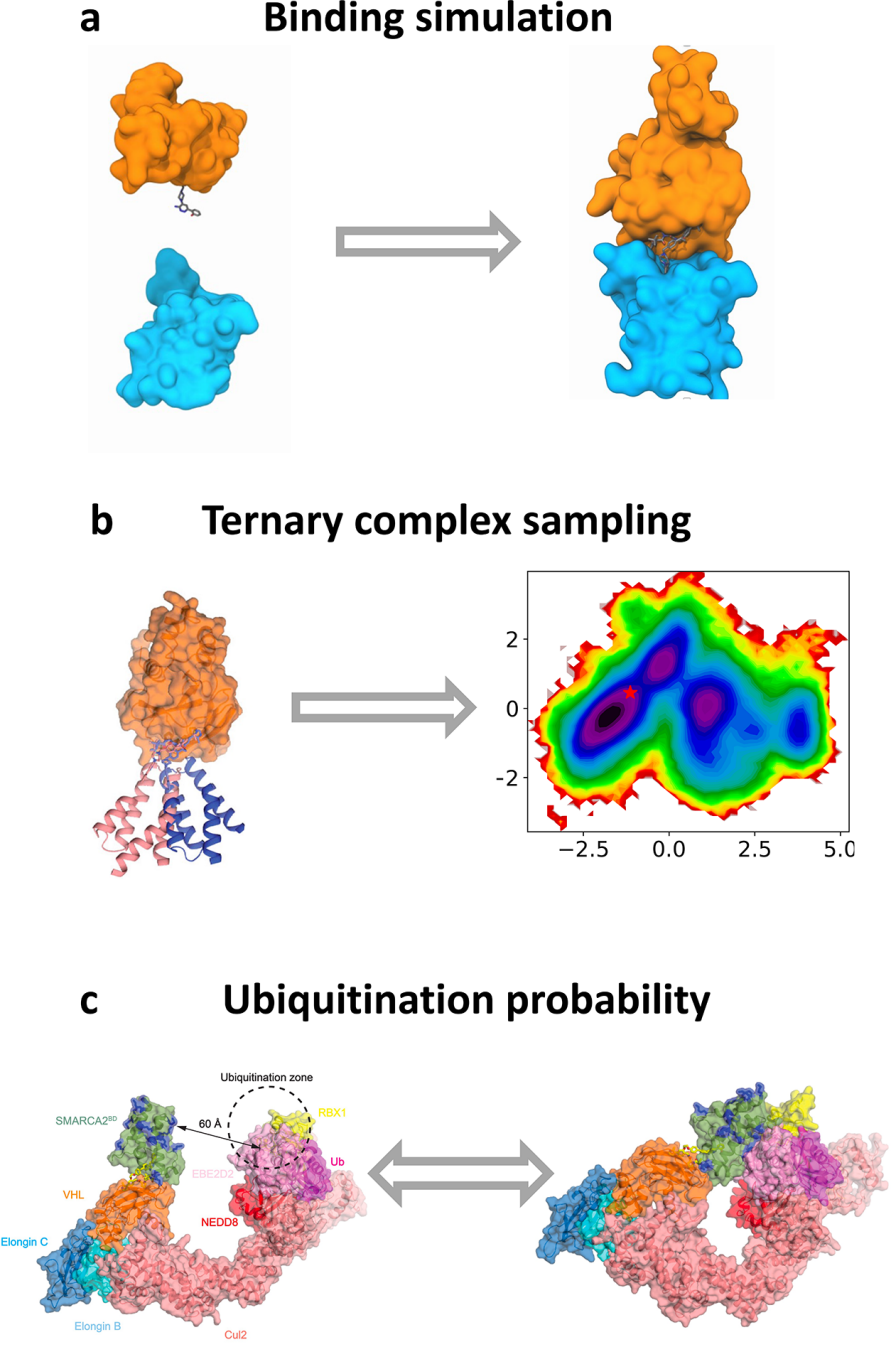
A simulation workflow to assess the quality of the degraders (illustrated here for the bromodomain of SMARCA2 as the target POI and VHL as the ligase). (a) The formation of ternary complexes is achieved by WE simulations, generating a variety of different ternary conformations. (b) Enhanced HREMD sampling helps explore ternary complex structures that may significantly differ from each other, producing a converged free energy surface. The star indicates the conformation of the crystal structure. (c) Analysis of ternary complex conformations in the context of the full CRL assembly, exhaustively sampled by meta-eABF, allows the assessment of the ubiquitination probability of the POI.

More rigorously, as illustrated in Figure 5, we have implemented a multistep simulation routine to assess the quality of degraders, that is, their ability to form a stable ternary complex with high ubiquitination probability. These simulations are applied to new degrader designs, but also to experimentally confirmed degraders and nondegrading heterobifunctional molecules, that, as discussed below, would then serve as input classes for a classifier to categorize a candidate molecule as a degrader or a nondegrader and also to guide the design of new degraders.

To drive the formation of ternary complexes (see Figure 5a), we apply weighted ensemble (WE) simulations starting with the well-separated POI and the ligase–degrader binary complex (or vice versa) and using as a collective variable the RMSD of only the warhead (or E3 ligand) to a corresponding bound structure of the other binary complex. The “Resampling of Ensembles by Variation Optimization” (REVO)^277^ is the WE algorithm of choice, as it yields ternary complexes with a variety of different conformations through the iterative optimization of “trajectory variation”.

Then, HREMD simulations are applied to bound ternary complexes using 20 replicas with an effective maximum temperature of 425 K, generating a more detailed map of the ensemble of ternary complexes (Figure 5b). We suggest to use a projection of the sampled conformations that epitomizes the interface, such as the principal components of site–site distance distributions.^108^ These conformational landscapes shed light on the stability of a ternary complex, and importantly, they can inform the docking scoring functions for the next round of designs.

Finally, to estimate a degrader’s impact on the ubiquitination of a POI, we superimpose the ternary complex structures obtained from HREMD on the full ubiquitin-bound Cullin–RING ligase (CRL) complex, which has been sampled exhaustively before between open and closed conformations by the metadynamics variant meta-eABF^238,239^ (Figure 5c). The distribution of distances between ubiquitin and different lysine residues on the target protein’s surface, computed over all frames, can be used as a scoring function regarding the propensity of ubiquitination induced by the simulated degrader. An alternative procedure that is also robust for predicting ubiquitination scores is to directly simulate the ternary complex in the CRL, rather than superimposing. Lysine distance profiles should be evaluated for each choice of simulation trajectory and model of the CRL.

We augment our modeling and simulation workflow for degrader design with a random forest classifier trained on known degraders and nondegraders of the target protein under consideration. Nondegraders are heterobifunctional molecules that have been experimentally verified to have very little or no degradation activity, which may be due to a variety of reasons. If available, experimental degradation data (e.g., *D*_max_) should be included in the model in addition to structural and physicochemical descriptors of the compound, such as the molecular weight and estimations of polarity and permeability, as well as features extracted from MD simulations of a fully solvated degrader that characterize its flexibility, like the (normalized) linker end-to-end distance or its gyration radius, and also such obtained from the ternary complex (HREMD) simulations, for instance, interface contacts and the lysine–ubiquitin distance distributions. We apply a principal component analysis to reduce the dimensionality in feature space and eliminate strong correlations among features. To train such a model for a relatively accurate classification of degrader candidates, we suggest to have a (well-balanced) set of at least 50 degrader/nondegrader molecules, to use two-thirds for training, and to cross-validate the ML model. Identifying the most predictive features tells us what input properties are crucial for degradation of the POI studied, which can strongly support the design process.

Our initial studies on designs of SMARCA2 degraders (described in more detail in Section 4.2) have revealed that the inclusion of all three feature categories, i.e., properties derived from the molecular structure, such obtained from fully solvated degrader simulations, and such from the ternary complex simulations, leads to classifiers with predictive ability of >80% accuracy, while those trained on only one of the mentioned categories are still sufficiently accurate (70–75%). Thus, to be operationally prudent, we suggest applying the random forest classifier to only the physicochemical and structural features of degrader designs in order to filter a smaller subset before running the more expensive simulation workflow and applying the ML model again, including features extracted from simulations.

Using 64 GPUs and about 500 CPUs, we were able to operate our design pipeline in 1 week, which is the appropriate time scale for decision making in a discovery program. The random forest models require only a few seconds for thousands or millions of virtual designs. Most of the computation is spent on docking of 500–1000 degrader candidates and MD simulations for the top 50 degrader compounds.

Although our design strategy features multiple modeling and simulation techniques, it must be complemented by experiments. Promising degrader candidates, i.e., those that have formed stable ternary complexes with a high ubiquitination probability and that have been labeled as a degrader by the ML classifier, must be tested by biochemical assays, e.g., by ALPHA or HiBiT, to attest their ability to form ternary complexes or degrade the target. This experimental feedback is instrumental in degrader design cycles, which, as mentioned earlier, are similar to any drug design cycle in that experimental expertise is necessary for decision making.

### 4.2 Application to a Degrader Design Project

**Figure 6:**
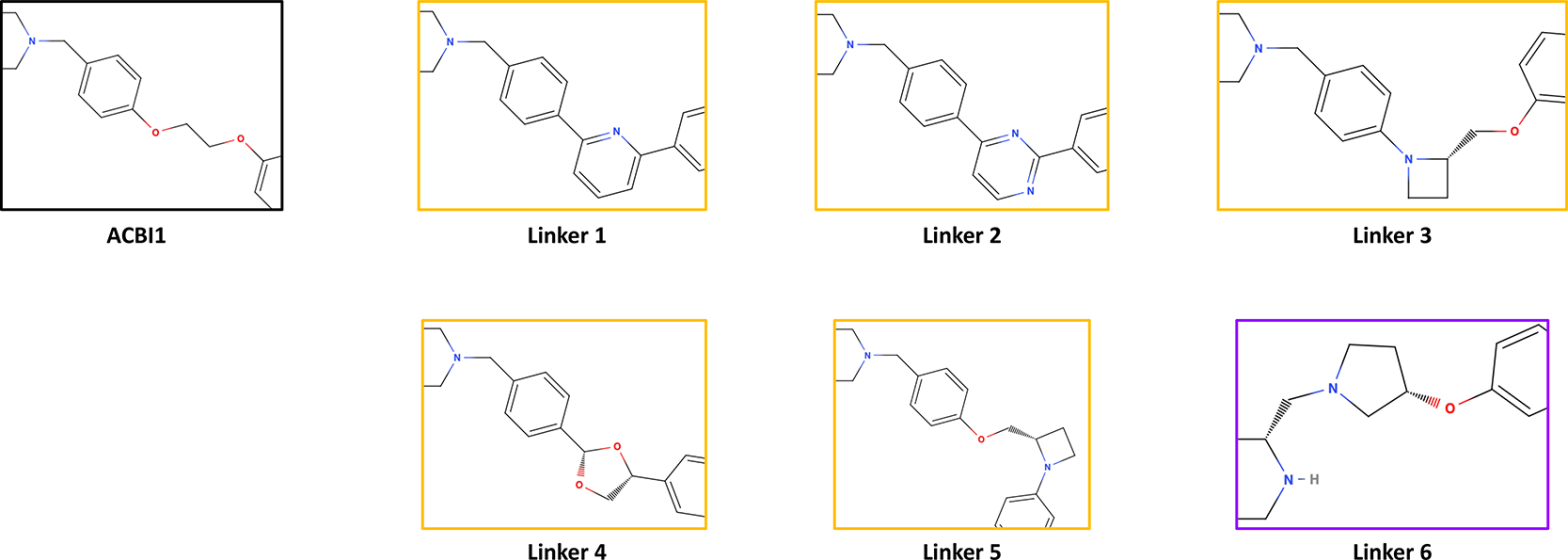
Linker designs. ACBI1 is the potent and cooperative SMARCA2 degrader optimized previously.^28^ Linkers 1–5 (orange frame) were shown to yield high SMARCA2 ubiquitination probabilities in simulations. Linker 6 (purple frame) was designed based on a metastable SMARCA2-VHL encounter complex.

**Figure 7:**
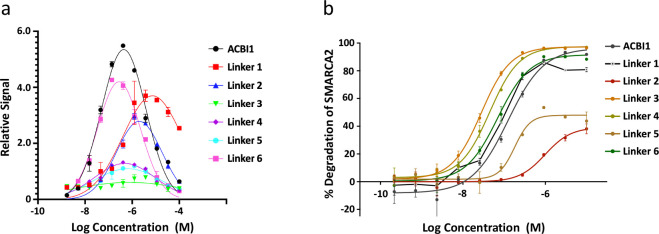
Experimental confirmation of degrader designs. (a) Fitted curves from AlphaLISA assays measuring ternary complex formation. (b) Degradation profiles from HiBiT assays.

We demonstrate the impact of computation by briefly presenting some results from our degrader discovery project, in which we designed heterobifunctional degraders for SMARCA2-VHL with a novel linker motif supported by our simulation workflow. Figure 6 lists all of our designs in this study (Linkers 1–6) along with ACBI1, a previously optimized VHL-recruiting degrader of SMARCA2.^28^ Informed by the random forest classifier, which was trained and validated on about 100 known compounds (model accuracy of 83%), the designs generally involve rigid linkers that contain (aromatic) ring structures with low molecular weight. Hence, five linkers were designed based on pyridine, pyrimidine, dioxolane, and azetidine heterocycles (Linkers 1–5 in Figure 6) with the objective of stabilizing the ternary complexes and improving the associated ubiquitination probability of lysine residues on the surface of SMARCA2. As shown in Table 2, three out of these five designs were found to have *D*_max_ > 85% and DC_50_ ≤ 80 nM in the corresponding experiments, validating the high degradation potency of these designs, and one of them had *D*_max_ = 54% and DC_50_ = 174 nM, which is still acceptable in terms of degradation activity (see experimental results in Figure 7). By contrast, 34 designs that incorporated typical alkyl and PEG linkers spanning 4 to ∼20 atoms as well as known linker moieties, such as triazoles and phenyl rings (*o*, *m*, or *p*-substituted to provide different geometries), only produced two active degraders.

**Table 2:** Summary of the Experimental Results on Degradation Activity of the Individual Linker Designs

Design	DC_50_ (nM)	*D*_max_ (%)	AlphaLISA (% of control)
ACBI1	37	94	100
Linker 1	80	86	69
Linker 2	n/a	38	52
Linker 3	27	97	11
Linker 4	43	97	20
Linker 5	174	54	20
Linker 6	67	90	78

**Figure 8:**
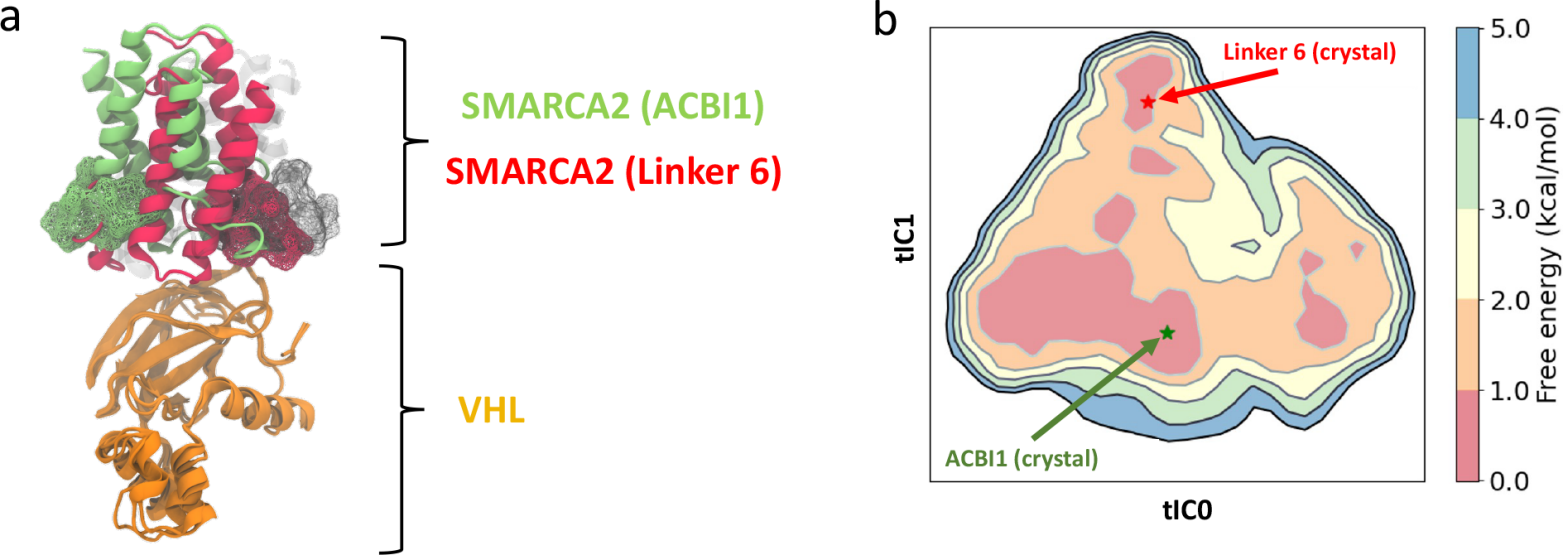
Comparison of ternary complexes with designed linkers. (a) Superposition of ternary complex crystal structures with Linker 6 (red SMARCA2 structure), designed in our study based on the simulation of SMARCA2-VHL encounter complexes, and with the previously designed ACBI1 degrader (green SMARCA2 structure).^28^ The structures are superimposed with the VHL ligases aligned. The transparent SMARCA2 structure is the model protein–protein encounter complex with which Linker 6 was designed. The contours indicate the electron densities of the corresponding warhead structures. (b) A free-energy landscape of the SMARCA2-VHL encounter complex conformations obtained from molecular simulations. tIC0 and tIC1 are the two slowest-relaxing degrees of freedom based on linear combinations of interface residue contacts between the two proteins observed during simulation as described elsewhere.^108^

Another demonstration of the impact of computation is the design of a SMARCA2-VHL degrader with a novel protein–protein interface. Briefly, about 150 SMARCA2-VHL conformations were generated from rigid protein–protein docking. 150 independent MD simulations of 3 μs were run starting at each one of these docked conformations to capture the baseline interactions between SMARCA2 and VHL. Markov state modeling revealed three main metastable states: one of the metastable states recapitulated the known crystal structures of SMARCA2-VHL ternary complexes (PDB IDs 6HAX, 6HAY, 74SE); another metastable state had a completely different protein–protein interface, in which SMARCA2 was indeed rotated by about 180° compared to the known crystal structures as illustrated in Figure 8. Using that latter state for structure-based design, we produced a relatively short linker motif including a pyrrolidine group and with attachment points different from those in the previously resolved crystal structures or in any of the past designs (Linker 6 in Figure 6). Our simulation workflow (see Figure 5) revealed that ternary complexes with this degrader candidate were relatively stable and SMARCA2 very likely to be ubiquitinated.

As displayed in Figure 8a, the crystal structure of the ternary complex with Linker 6 (red structure) is similar to the corresponding simulated structure (transparent structure), based on which the degrader was designed, thus validating this approach of a simulation-driven structure-based degrader design. Furthermore, Figure 8a confirms that this degrader induces an obviously different conformation compared with the previously known ternary complex crystal structure with ACBI1 (green structure). Despite the distinctly different conformations, the interface–RMSD of these two ternary complexes is 2.6 Å and about 40% of the same interface atoms are in contact between the two corresponding crystal structures. Figure 8b visualizes a free energy landscape of the SMARCA2-VHL encounter complex simulations that capture all baseline interactions of this POI–ligase pair. Obviously, the ternary complex crystal structures with ACBI1 and Linker 6 occupy different metastable states. The degrader candidate based on Linker 6 was classified as a degrader by our ML model, which has been confirmed experimentally with *D*_max_ = 90% and DC_50_ = 67 nM.

The results presented for the SMARCA2-VHL degrader design underscore the suitability and the benefit of the modeling and simulation strategies we developed. In particular, the prediction of a favorable, previously unknown, protein–protein interface, which served as a template to design an active degrader, is, in our opinion, a remarkable achievement, showing the potential of a simulation-driven protocol and its complementarity to experiments. Importantly, we believe that our research has highlighted how computational methods, similar to experiments, permit us to approach the task of degrader design from different angles. We have combined results from simulations that examine POI–ligase interactions with such data that assess the stability of ternary complexes and its ubiquitination probability in the context of a supramolecular protein aggregate, showing how the integration of multiple molecular simulation (and docking) methods can support degrader design.

We envision that in the future, a variety of different computational protocols would be applied for TPD analysis and degrader design. Depending on the POI–ligase system and the availability of data on known active degraders, we anticipate that the focus might lie on predicting only distinct features that could complement experiments. For instance, these may be the likelihood of certain lysines being ubiquitinated in ternary complexes or certain regions on the protein surfaces being solvent-exposed, which can be assessed by the simulation methods discussed. Also, if the rapid screening of designs is required for decision making, ternary complex docking, followed by short MD simulations, will remain the primary approach. However, as our research results demonstrate, a rigorous strategy to examine degrader designs and explore protein–protein interactions is often necessary and, evidently, achievable.

## 5 Future Directions

This is a unique time in the field of targeted protein degradation (TPD). After decades of academic research, we are now seeing biotechnology and pharmaceutical companies bring TPD molecules to the clinic. While it appears that computational tools were used in the design of some clinical-stage molecules, their impact was, in fact, minimal. However, there is a growing number of examples where computational approaches are being used to address some of the great challenges in TPD drug discovery.

As discussed in this Perspective, there is a diverse array of computational tools contributing to the TPD field. In some cases, traditional tools can be easily repurposed from small molecule applications to TPD. For example, docking and screening of warheads and E3 ligands are analogous to small molecules and can therefore be used directly. Similarly, many of the underlying methods to predict properties are the same (e.g., QSAR and machine learning based on experimental data), although the data are quite sparse and the chemical space is much larger for many TPD molecules, making the existing models less predictive. In other cases, traditional tools require additional training and parameter tuning to improve results for TPD, such as protein–protein docking algorithms, where constraints related to the binding sites and warhead/ligand attachment points can significantly reduce the search space, thereby improving both the speed and accuracy of the algorithms.

Perhaps the most interesting, innovative, and impactful is the growing number of approaches to simulate the dynamic behavior of the ternary complex. The significance of these methods lies in the fact that the formation of a ternary structure is a necessary step in the TPD process and, furthermore, that the non-native protein–protein interactions seem to be “floppier” than many endogenous protein–protein complexes. Indeed, biology is in constant motion, and not surprisingly, TPD follows the same paradigm. The importance of understanding the induced ternary structures stems from the criticality of this step in the degradation process, where the ubiquitination mechanism requires not just binding but also forming the correct orientation of the protein of interest in relationship to the rest of the supramolecular assembly that is responsible for the ubiquitination. This is supported by a growing body of evidence, both computational and experimental.

In addition to dynamics and conformational variability in the ternary complex, early works that simulate the full supramolecular complex (e.g., the Cullin–RING ligase) leading to ubiquitination of the protein of interest have yielded promising results and significant insights into the TPD process. These simulations involve hundreds of thousands of atoms and therefore require significant computational time and resources; fortunately, these simulations will become more accessible as computer hardware continues to grow in power, efficiency, and affordability. Still, for years to come, brute force simulations of meaningful time scales for TPD will be out of reach for most researchers and restricted to specialized hardware like the Anton supercomputer^278^ or massively distributed systems like Folding@home.^279^ Fortunately, enhanced sampling algorithms can greatly accelerate simulations, especially when there is knowledge about the collective variable (CV) of interest. In the case of the Cullin–RING Ligase (CRL), there is a growing body of biophysical and structural biology data that facilitates the elucidation of practical CVs, as described in this Perspective.

Aside from the computational approaches discussed here, we are seeing many other technology advances related to TPD. Structural biology techniques like cryogenic electron microscopy (cryo-EM) have enabled atomic-resolution structures for supramolecular protein assemblies like the CRL. Still, structure (even with dynamics) is insufficient to design drugs. When developing degrader compounds, it is important to optimize additional properties, such as cellular permeability and affinity. Approaches such as the NanoBRET target engagement assay provide a quantification of interactions in live cells, which encapsulates permeability, affinity, and residence time. Mass spectrometry-based chemoproteomics is also enabling TPD discovery efforts, from screening for chemical scaffolds that bind to proteins in their native environment, including post-translational modifications, to characterizing the time-dependent degradation process in the cell. Additionally, as more high-content experimental information becomes available, such as live-cell kinetic data from technologies like HiBiT, the ability to predict and improve degradation profiles becomes more amenable to advanced ML algorithms such as 4D equivariant graph transformer representations of simulated ternary complex molecular dynamics. This specific approach encodes MD trajectories of ternary complexes as graphs that are transformed and used for the training of feed-forward networks to predict the functional form defined by the raw HiBiT data over time, enabling the calculation of pharmacological constants such as DC_50_, *D*_max_, and the degradation rate. These approaches and other experimental data can be used in many ways to improve our understanding of the TPD process, such as building mathematical models to connect quantities that can be more readily computed (e.g., permeability, affinity, and stability of the ternary complex) to important downstream processes (e.g., degradation efficiency).^266^

The integration of the computational and experimental methods introduced in this Perspective is the key to success in TPD research. In our opinion, which is based on the state-of-the-art methods and the promising results presented here, there are three main avenues of combination: first, experimentally generated data on degrader physicochemical, binding, and activity properties support predictive models; second, structural proteomics and biophysics enhance docking and simulation procedures; and third, monitoring the degree of ternary complex formation and target degradation is necessary for computation-enabled degrader design cycles. This level of interdependence between experiments and computation calls for a coordinated community-wide effort. However, to bring to fruition the promise of proteasomal degradation as a therapeutic modality, collaborations must go beyond simple information exchange; rather, they must comprise multidisciplinary research teams dedicated to exploring the different facets of TPD. Specifically, for the design of degrader candidates, as we outlined above, simulation or data mining results must be translated and implemented upon careful deliberation with synthetic and medicinal chemists. While such teamwork has always been required in drug discovery settings, it is all too often neglected. The intriguing task of developing heterobifunctional degrader molecules with high specificity and potency shows quite plainly how the cooperation between both modelers and experimentalists could lead to sustained productivity.

While much progress has been made in the TPD field, more work is needed in our quest to more efficiently design more effective TPD therapeutics. First, traditional QSAR models generally perform poorly for large heterobifunctional degrader molecules. A combination of more experimental data coupled with improved QSAR modeling approaches (perhaps accounting for 3D or even dynamic information) is likely required. Improved docking and structure prediction methods would also be of significant value. Ternary complex docking is a relatively new problem, and most early applications have involved retrofitting existing tools. It is likely that new algorithms, built from the ground up, will be better at solving this specific problem. Finally, molecular design tools for the TPD are greatly needed. Repurposing tools like DeLinker^213^ in our experience has provided some value, but there are still significant gaps in terms of designing degraders that can be readily synthesized and have good ADMET properties.

When developing new tools, the broad direction of the TPD field should be considered. Most early work focused on degradation through inducing proximity to enable ubiquitination. In this context, covalently binding degraders are considered to be more efficacious.^219,280^ Also, many of the approaches, that we presented in this Perspective on heterobifunctional degraders, can be applied to the characterization of molecular glues, which, however, presents its own set of challenges, such as the screening for suitable chemical matter or the identification of an appropriate E3 ligase for a given target POI. Moreover, we are now seeing the surfacing of many other modes of degradation, such as lysosome-targeting chimeras (LYTACs^281^), macroautophagy degradation targeting chimeras (MADTACs^282^), autophagy-targeting chimeras (AUTOTACs^283^), deubiquitinase-targeting chimeras (DUBTACs^284^), and chaperone-mediated protein degraders (CHAMPs^285^). Additionally, induced proximity is being leveraged for nondegradation applications, such as phosphorylation-inducing chimeric small molecules (PHICS^286^), where a small molecule brings a kinase into proximity with a protein of interest to phosphorylate the target protein, which is not otherwise a substrate for the kinase.

We see a bright future for the field of TPD, and induced proximity approaches more broadly, with encouraging clinical data emerging and a growing number of innovative companies in the preclincial stage. We envision continued improvements across many different computational and experimental methods that will contribute to a rich ecosystem that companies will leverage to build integrated workflows that solve some of the most critical challenges in the field. Ultimately, with the confluence of advances in different methods, we expect to see the emergence of next-generation workflows that will show demonstrable advantages over approaches that do not leverage computation. The greatest successes will come from the computational efforts that are tightly integrated with advanced experimental approaches in an iterative fashion. We hope that our Perspective will spark some ideas and contribute to the future of the exciting field of targeted protein degradation.

## Glossary

**Targeted Protein Degradation (TPD)**: A biomedical research strategy that aims to selectively eliminate specific proteins from cells for therapeutic applications, often using heterobifunctional degraders or molecular glues.
**Heterobifunctional**: A molecule or agent with different structural motifs (or functional groups), enabling simultaneous interaction with multiple biological targets.
**Proteasome**: A protein complex that degrades unneeded or damaged proteins by proteolysis, a chemical reaction that breaks peptide bonds.
**Degrader**: A heterobifunctional molecule designed to induce TPD, bridging a specific protein of interest with an E3 ubiquitin ligase to trigger proteasomal degradation (often referred to as a PROteolysis TArgeting Chimera or PROTAC).
**E3 ligase**: An enzyme that plays a crucial role in the ubiquitin–proteasome system, catalyzing the transfer of ubiquitin to specific target proteins, thereby marking them for degradation.
**Protein of interest (POI)**: The target protein to be eliminated through the TPD process.
**Warhead**: The structural motif of a heterobifunctional degrader that is designed to bind a specific protein of interest and to induce its degradation.
**E3 ligand**: A small-molecule ligand of the E3 ligase that facilitates its interaction with a target protein.
**Linker**: A structural motif that connects the warhead to the E3 ligand within a heterobifunctional degrader, facilitating proximity and interaction for targeted protein degradation.
**Hook effect**: Also known as the prozone effect, it refers to the phenomenon in which the efficacy of bivalent molecules, like heterobifunctional degraders, decreases at high concentrations due to the increased formation of unproductive (binary) complexes.
**Cooperativity**: A phenomenon in biochemical systems where the binding of a molecule to a site on a protein affects the affinity of subsequent molecules binding to additional sites on the same protein, leading to either enhanced (positive cooperativity) or reduced (negative cooperativity) binding.
**DC_50_**: A term used in TPD to denote the concentration of a degradation-inducing agent required to achieve 50% of its maximum degradation capacity for a specific POI.
**D_max_**: The maximum degradation capacity of a TPD-inducing agent representing the highest achievable level of POI-degradation under given conditions.
**Beyond Rule of 5 (bRo5)**: Compounds, like heterobifunctional degraders, that violate Lipinski’s ‘Rule of 5’, which predicts the success of orally absorbed drugs, yet can be effective therapeutics.
**Chameleonicity**: The ability of a molecule to expose polar groups in aqueous environments and, through conformational changes, bury them during membrane permeation thus contributing to its bioavailability and pharmacological profile.
**Ternary complex docking**: A computational approach to study the formation and stability of a complex involving three molecular entities, such as a POI and an E3 ligase connected by a degrader.
